# A systematic review on the accumulation of prophylactic dosages of low-molecular-weight heparins (LMWHs) in patients with renal insufficiency

**DOI:** 10.1007/s00228-015-1880-5

**Published:** 2015-06-14

**Authors:** Ferdows Atiq, Patricia M.L.A. van den Bemt, Frank W.G. Leebeek, Teun van Gelder, Jorie Versmissen

**Affiliations:** Department of Hospital Pharmacy, Erasmus Medical Center, Rotterdam, The Netherlands; Department of Hematology, Erasmus Medical Center, Rotterdam, The Netherlands; Department of Internal Medicine, Erasmus Medical Center, PO Box 2040, 3000 CA Rotterdam, The Netherlands

**Keywords:** Clinical trials, Heparins, Pharmacodynamics, Heparins, Venous thrombosis

## Abstract

**Purpose:**

Although therapeutic dosages of most low-molecular-weight heparins (LMWHs) are known to accumulate in patients with renal insufficiency, for the lower prophylactic dosages this has not been clearly proven. Nevertheless, dose reduction is often recommended. We conducted a systematic review to investigate whether prophylactic dosages of LMWH accumulate in renal insufficient patients.

**Methods:**

A comprehensive search was conducted on 17 February 2015 using Embase, Medline, Web of Science, Scopus, Cochrane, PubMed publisher, and Google scholar. The syntax emphasized for LMWHs, impaired renal function, and pharmacokinetics. The search yielded 674 publications. After exclusion by reading the titles, abstracts, and if necessary the full paper, 11 publications remained.

**Results:**

For dalteparin and tinzaparin, no accumulation was observed. Enoxaparin, on the other hand, did lead to accumulation in patients with renal insufficiency, although not in patients undergoing renal replacement therapy. Bemiparin and certoparin also did show accumulation. No data were available for nadroparin.

**Conclusions:**

In this systematic review, we show that prophylactic dosages of tinzaparin and dalteparin are likely to be safe in patients with renal insufficiency and do not need dose reduction based on the absence of accumulation. However, prophylactic dosages of enoxaparin, bemiparin, and certoparin did show accumulation in patients with a creatinine clearance (CrCl) below 30 ml/min, and therefore, dose reduction is required. The differences in occurrence of accumulation seem to depend on the mean molecular weight of LMWHs.

**Electronic supplementary material:**

The online version of this article (doi:10.1007/s00228-015-1880-5) contains supplementary material, which is available to authorized users.

## Introduction

Low-molecular-weight heparins (LMWHs) are anticoagulants made by depolymerization of unfractioned heparin (UFH) [1, 2]. In the last decades, LMWHs have largely replaced UFH as anticoagulants, because they are at least equally effective in prevention and treatment of venous thromboembolisms (VTEs) and have many practical advantages, most importantly the possibility of subcutaneous administration without the need of routine laboratory monitoring of the anticoagulant response [3–8]. Moreover, LMWHs have a more predictable anticoagulant response, longer half-life (allowing once or twice daily administration), and a dose-independent elimination and they cause less heparin-induced thrombocytopenia [1, 2, 9, 10]. There is conflicting evidence whether bleeding complications differ between LMWHs and UFH, although a Cochrane analysis showed that LMWHs reduce the occurrence of major bleedings [8, 11–14].

The molecular weights of the various LMWHs differ. Tinzaparin has the highest average molecular weight (6500 Da), while certoparin has the lowest average molecular weight (3800 Da) [13]. Pharmacokinetics of different LMWHs vary: they differ in elimination half-life, clearance, and bioavailability [15, 16]. This might be a consequence of the differences in molecular weight. Also with regard to pharmacodynamics, LMWHs vary: anti-Xa/anti-IIa activity ratios range between 1.5 and 2.5 (tinzaparin and certoparin) to 3.6–6.5 (reviparin) [13].

Since LMWHs are mainly excreted by the kidney, they may accumulate in patients with renal insufficiency increasing the risk of bleeding [17–19]. Due to above-described differences in pharmacokinetics, data on accumulation in renal insufficiency cannot be easily converted from one LMWH to another [2]. For instance, therapeutic dosages of different LMWHs do not all accumulate in patients with renal insufficiency: nadroparin and enoxaparin were found to accumulate, while tinzaparin did not [20–26]. For LMHWs that accumulate in a therapeutic dosage, dose reduction is often recommended. Also for the lower prophylactic dosages, such dose reductions have been suggested, especially for high prophylactic dosages such as those used in cancer patients or in high-risk surgery [24, 27–29].

Even in this era of novel oral anticoagulants, LMWHs will stay important for the initial treatment of VTE during the first 5–7 days in patients treated with dabigatran and edoxaban, as well as for prophylaxis of VTE. Especially for patients with severe renal insufficiency in which case the novel oral anticoagulants are contraindicated, it remains important to know whether LMWHs can be prescribed safely.

Therefore, we conducted a systematic review to investigate whether prophylactic dosages of various LMWHs lead to accumulation defined as an increase in anti-Xa activity and whether accumulation depends on molecular weight of the LMWH.

## Materials and methods

The meta-analysis was prepared in accordance with the PRISMA (Preferred Reporting Items for Systematic Reviews and Meta-Analyses) statement [30]. No prespecified formal protocol was registered.

### Article search

A comprehensive, systematic literature search has been conducted on 17 February 2015 using Embase, Medline, Web of Science, Scopus, Cochrane, PubMed publisher, and Google scholar. The syntax emphasized for LMWHs, impaired renal function, and pharmacokinetics using synonyms and relevant terms. The search terms as used in Embase, as an example, are shown in a supplemental file. In addition to the search results, we manually searched reference lists of all relevant articles for additional studies.

### Inclusion criteria and exclusion criteria (eligibility criteria)

We only included articles in English on prophylactic LMWH treatment, studying at least ten patients (non-pregnant and with no children) with renal impairment. We defined accumulation as an increase in anti-Xa activity after consecutive administration for several days. Therefore, we excluded studies which did not administer LMWH on consecutive days (except for studies in patients receiving renal replacement therapy) and did not measure anti-Xa activity on multiple days while giving LMWH. We excluded case reports, overviews, expert opinions, recommendations, reviews, and replies on articles. Abstracts of unpublished data were not excluded; authors were approached for additional information.

### Study selection and data collection

A single reviewer excluded articles that did not meet the eligibility criteria by using the title and abstract. If necessary, the full text was read. If the reviewer could not decide whether to exclude the article, a second reviewer was asked for advice and both met to reach a consensus. Furthermore, a single reviewer collected relevant data from the included articles for the review.

### Risk of bias

Risk of bias was evaluated at study level using the Cochrane Risk of Bias assessment tool. The criteria on random sequence generation, allocation, and blinding were disregarded.

## Results

### Study selection

**Fig. 1:**
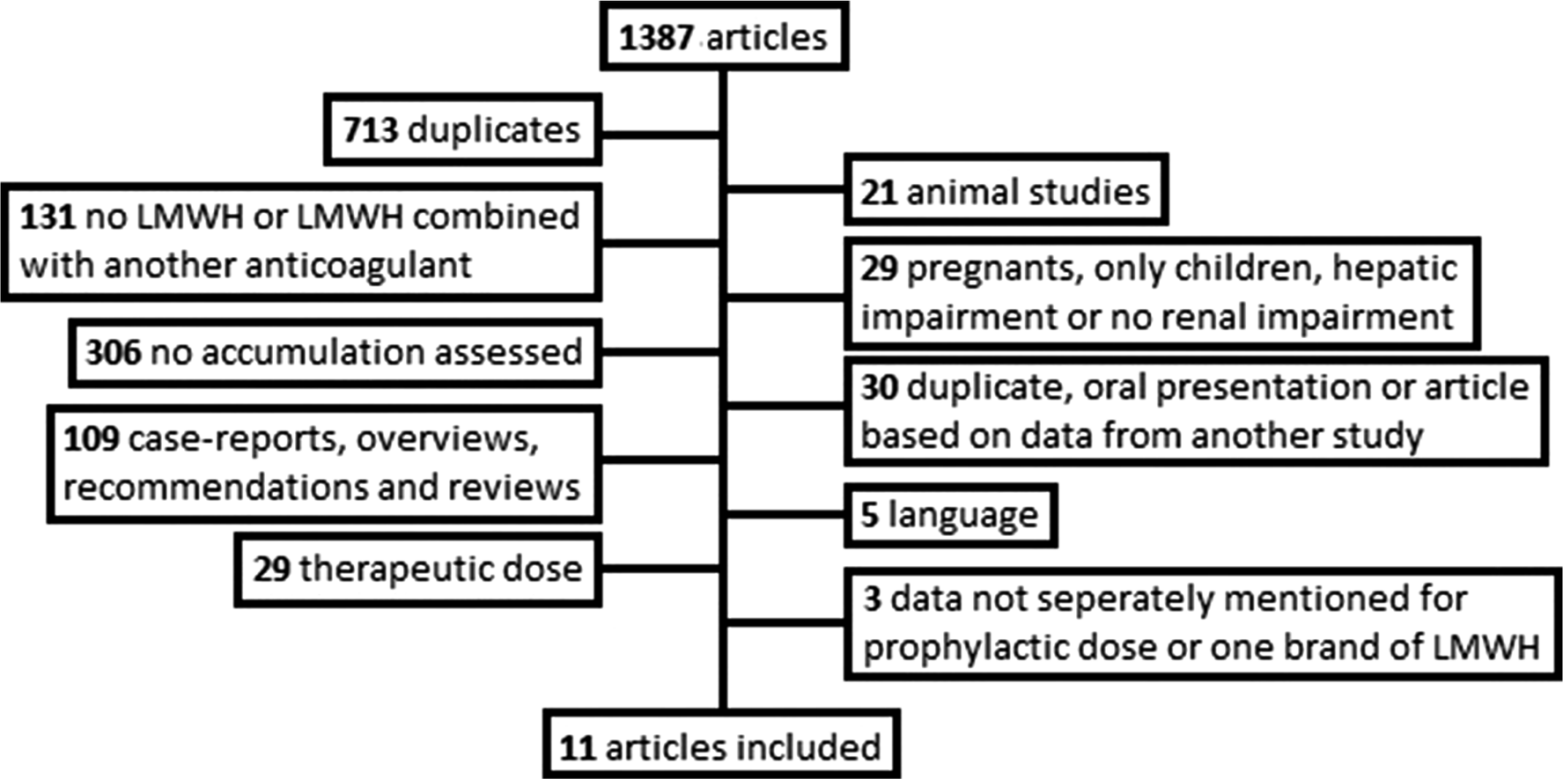
Flow diagram illustrating literature evaluation

The search yielded 1387 articles from which 713 were duplicates. Figure 1 shows a flow diagram illustrating literature evaluation.

### Characteristics

Table 1 shows the study and patient characteristics. We included 11 articles of which one was on LMWH in continuous venovenous hemofiltration (CVVH) and one on hemodialysis patients. Five studies examined dalteparin accumulation, and five articles examined enoxaparin [31–37, 40, 41]. For tinzaparin, bemiparin, and certoparin, there was one study for each LMWH [35, 38, 39].

**Table 1:** Study characteristics of included studies including patient characteristics and outcome measurements

Study	Study design	LMWH	Patients (*n*)	Groups	Patient characteristics	Follow-up	Outcomes
Rabbat 2005^a^ [31]	Cohort	Dalteparin 5000 IU	19	NA	IC patients with CrCl ≥ 30	12 days^f^ (IQR 8–24)	Trough (22–23 h postinjection) and peak anti-Xa
Schmid 2009^b^ [32]	Cohort	Dalteparin <50 kg 2500 IU 50–100 kg 5000 IU >100 kg 7500 IU	42	*n* = 18 CrCl ≥ 60 *n* = 15 CrCl 30–59 *n* = 9 CrCl < 30	General medical and surgical ward	3 weeks	Peak anti-Xa
Douketis 2008^a^ [33]	Cohort	Dalteparin 5000 IU	138	NA	ICU patients with CrCl < 30 ml/min	9 days^f^ (IQR 5–15)	Trough (20 h postinjection) and peak anti-Xa
Tincani 2006^b^ [34]	Cohort	Dalteparin High risk 5000 IU Low risk 2500 IU	115	*n* = 9 CrCl 60–89 *n* = 73 CrCl 30–59 *n* = 24 CrCl < 30	Elderly patients (>65 years) with acute illness	6 days	Peak anti-Xa
Mahe 2007^a^ [35]	Randomized	Tinzaparin 4500 IU Enoxaparin 4000 IU	50	*n* = 27 tinzaparin; *n* = 28 enoxaparin	CrCl 20–50 ml/min, >75 years and weight < 65 kg	8 days	*C*_max_, AUC, and trough anti-Xa (24 h postinjection)
Mahe 2007^a^ [36]	Cohort	Enoxaparin 4000 IU	125	CrCl 51–80 CrCl 41–50 CrCl 31–40 CrCl 20–30	Elderly patients (>75 years) with acute medical illness	10 days	Peak anti-Xa. Maximum value per patient was used in the analysis
Sanderink 2002^c^ [37]	Cohort	Enoxaparin 4000 IU	48 (12/group)	CrCl > 80 50 < CrCl ≤ 80 30 < CrCl ≤ 5 CrCl ≤ 30	18–75 years old. BMI 18–30	4 days	*C*_max_, AUC, half-life, and CL/F
Rico 2014^d^ [38]	2-period cohort	Bemiparin 3500 IU	48	*n* = 25 CrCl > 80 *n* = 8 50 ≤ CrCl ≤ 80 *n* = 7 30 ≤ CrCl < 50 *n* = 8 CrCl < 30	Persons with normal renal function had no medical history	4 days	*C*_max_, AUC, half-life, and CL/F
Alban 2013^e^ [39]	Cohort	Certoparin 3000 IU	24	*n* = 12 normal renal function *n* = 12 CrCl < 30		5 days	*C*_max_, AUC, half-life
Polkinhorne 2002^e^ [40]	Randomized	Enoxaparin 4000 IU Dalteparin 2500 IU	21 HD	Each drug *n* = 7	Thrice-weekly hemodialysis patients	4 weeks	Anti-Xa activity 1, 2, 3, 4, 24, and 28 h postinjection
Brown 2010^e^ [41]	Cohort	Enoxaparin 3000 IU	12 CVVH	NA	ICU patients receiving CVVH 30 ml/kg/h	9 days^g^	Trough anti-Xa levels

All studies administered a once daily dosage. Peak anti-Xa: activity 4 h postinjection in all studies

CrCl was estimated using the ^a^Cockroft Gault formula; ^b^MDRD formula; ^c^formula: (urine creatinine (mg/dl) × 24 h urine volume (ml)/1440 min)/(Ʃ[serum creatinine from days 1, 4, and 5 (mg/dl)]/3); ^d^formula: urine creatinine concentration × 24 h collected urine volume/plasma creatinine concentration × 24 × 60. ^e^CrCl formula is not mentioned. ^f^Median length of stay. ^g^Time between first and last anti-Xa in whole population

*ICU* intensive care unit, *CrCl* creatinine clearance in ml/min, *IQR* interquartile range, *n* number of patients, *C*_*max*_ maximum concentration, *AUC* area under the curve, *BMI* body mass index, *CL*/*F* apparent total body clearance, *HD* hemodialysis, *CVVH* continuous venovenous hemofiltration

All studies were conducted prospectively. Two were randomized trials, and eight were cohort studies. Only one study did report clear prespecified primary outcomes. The number of patients ranged between 12 and 138. Three studies only included patients on an intensive care unit (ICU), and three studies only included elderly patients (Table 1). The follow-up length per patient was 4 days to 3 weeks. Accumulation was mainly assessed by measuring peak anti-Xa activity or trough anti-Xa activity (Table 2). Six studies reported clinical outcomes like hemorrhagic events, thrombosis, and mortality rates.

**Table 2:** Outcomes of included studies

LMWH	Parameter	Results					
Dalteparin 5000 IU [31]	Trough anti-Xa	Three patients value(s) > detection threshold; none above accumulation threshold					
	Peak anti-Xa	Mean 0.30 U/ml (95 % CI 0.27–0.33)					
		Day 1			Day 10		
Dalteparin 2500, 5000, and 7500 IU [32]		CrCl > 60	CrCl 30–59	CrCl < 30	CrCl > 60	CrCl 30–59	CrCl < 30
	Peak anti-Xa (range)	0.28^b^ (0.20–0.32)	0.31^b^ (0.23–0.46)	0.28^b^ (0.23–0.33)	0.27^a,b^ (0.16–0.35)	0.48^a,b^ (0.31–0.51)	0.39^a,b^ (0.31–0.50)
Dalteparin 5000 IU [33]	Trough anti-Xa	Seven patients value(s) > detection threshold; none above accumulation threshold					
		Day 3		Day 10		Day 17	
	Peak anti-Xa (range)	0.29 (0.20–0.42)^a^		0.35 (0.24–0.43)^a^		0.34 (0.27–0.45)^a^	
Dalteparin 2500 and 5000 IU [34]		Day 6					
		CrCl > 60		CrCl 30–59		CrCl < 30	
	Peak anti-Xa (range)	0.030 (0.086)^b^		0.033 (0.075)^b^		0.048 (0.084)^b^	
		Day 1		Day 8		*p* value	
Enoxaparin 4000 IU Tinzaparin 4500 IU [35]		Enoxaparin	Tinzaparin	Enoxaparin	Tinzaparin	Enoxaparin	Tinzaparin
	*C*_max_	0.55 (0.14)	0.44 (0.16)	0.67 (0.23)	0.46 (0.19)	<0.001	0.296
	AUC	354 (119)	252 (103)	447 (218)	273 (111)	<0.001	0.11
	Trough	0.06 (0.06)	0.05 (0.04)	0.11 (0.10)	0.06 (0.06)	0.013	0.17
Enoxaparin 4000 IU [36]		CrCl 51–80	CrCl 41–50	CrCl 31–40	CrCl 20–30		
	Anti-Xa_max 1–10_	0.60 (0.16)	0.61 (0.17)	0.61 (0.24)	0.72 (0.27)		
	*p* value	0.030^c^	0.039^c^	0.039^c^	Ref		
Enoxaparin 4000 IU [37]		Severe RI					
	*C*_max_	10–35 % higher					
	AUC_(0–24)_	65 % higher (day 4)					
	CL/F	27 % (day 1), 39 % (day 4)					
	*t*_1/2λz_	Increased with the degree of RI (*p* < 0.012)					
Bemiparin 3500 IU [38]		Severe RI					
	*C*_max_	Higher					
	*t*_1/2_	2–4 h prolonged					
	CL/F	Lower					
	AUC	Increased with the degree of RI					
Certoparin 3000 IU [39]		Severe RI			Ratio severe RI/normal renal function		
	*C*_max_ day 5	0.27 (range 0.16–0.70)			1.39 (95 % CI 1.04–1.85)		
	AUC_(0–24)_	2.28 (range 1.35–5.11)			1.52 (95 % CI 1.07–2.17)		
Enoxaparin 4000 IU, Dalteparin 2500 IU [40]		Week 1^a^			Week 4^a^		
		Dalteparin	Enoxaparin		Dalteparin	Enoxaparin	
	Anti-Xa (SEM)	0.2 (0.035)	0.38 (0.028)		0.26 (0.038)	0.40 (0.055)	
Enoxaparin 3000 IU [41]	Trough anti-Xa	Mean 0.11 (range 0.01–0.27, SD 0.07). None above accumulation threshold.					

All studies administered a once daily dosage. Peak anti-Xa: activity 4 h postinjection in all studies (all anti-Xa activity in IU/ml

*IQR* interquartile range, *CrCl* creatinine clearance in ml/min, *C*_*max*_ maximum concentration, *AUC* area under the curve, *Anti*-*Xa*_max 1–10_ maximum anti-Xa activity in 10 days, *AUC*_(0–24)_ area under the 24 h plasma activity time curve, *CL*/*F* apparent total body clearance, *RI* renal insufficiency, *t*_1/2_ elimination half-life, *t*_1/2λz_ apparent terminal elimination half-life, *SEM* standard error of the mean

^a^No significant changes between day 1 and day *x*

^b^No significant changes between groups on day *x*

^c^Compared to CrCl 20–30 ml/min

### Dalteparin

Dalteparin accumulation was studied in five articles. In patients on a general medical or surgical ward, no accumulation was found in a total of 157 patients by measuring peak anti-Xa activity on day 6 or day 10 [32, 34].

At the ICU, trough and peak anti-Xa activity did not show accumulation on day 9 or day 12 in a total of 157 patients not undergoing CVVH [31, 33].

Also in hemodialysis patients (*n* = 7) prescribed 2500 IU dalteparin during hemodialysis sessions for 4 weeks, anti-Xa activity at different time points (1, 2, 3, 4, 24, and 28 h postinjection) were not significantly different between week 1 and week 4 [40]. The patients underwent hemodialysis sessions three times a week for 4 h.

### Enoxaparin

Three studies of which two in patients older than 75 years examined enoxaparin accumulation in, respectively, 125, 28, and 48 non-dialysis patients [35–37]. The studies in the elderly with CrCl 20–50 ml/min found a significantly higher maximum concentration (*C*_max_), area under the curve (AUC), and trough anti-Xa activity (24 h postinjection) after 8 days administration and a higher peak anti-Xa activity after 10 days, compared to patients with better renal function [35, 36]. The other study in patients with different severity of renal insufficiency found accumulation (a significantly higher *C*_max_, longer half-life, higher AUC) already on day 4 in patients with CrCl ≤ 30 ml/min [37].

Hemodialysis patients (*n* = 7) prescribed 40 mg enoxaparin at hemodialysis sessions for 4 weeks, anti-Xa activity at different time points postinjection were not significantly different between week 1 and week 4 [40]. Also in ICU patients (*n* = 12) undergoing CVVH with a flow rate of 30 ml/kg/h, no accumulation of enoxaparin (30 mg daily) was found [41].

### Tinzaparin, bemiparin, and certoparin

Administration of 4500 IU tinzaparin for 8 days in 27 patients older than 75 years with CrCl 20–50 ml/min and body weight below 65 kg did not lead to significant changes in *C*_max_, AUC, and trough anti-Xa activity (24 h postinjection) [35].

For 3500 IU bemiparin, *C*_max_ was higher and mean half-life was 2–4 h prolonged in patients with severe renal insufficiency after 4 days administration in 48 patients [38]. AUC significantly increased with the degree of renal insufficiency.

For 3000 IU certoparin, *C*_max_ and AUC were significantly higher in patients with renal insufficiency compared to healthy controls after 5 days administration in 24 patients [39].

### Adverse events

Six studies reported VTEs and bleeding events, although all were underpowered to find significant correlations [31–36]. In three of these studies, anti-Xa activity was undetectable during bleeding [31, 33, 34]. Five serious bleeding complications were reported when using enoxaparin, but anti-Xa activity in these patients was the same as in those without bleeding (*p* = 0.77) [36].

## Discussion

In this systematic review, we show that prophylactic dosages of dalteparin and tinzaparin did not accumulate in patients with renal insufficiency, while prophylactic dosages of enoxaparin, bemiparin, and certoparin did accumulate. Dalteparin also showed no accumulation in hemodialysis patients. Surprisingly, enoxaparin did not show accumulation in hemodialysis and CVVH patients, which might be due to removal by renal replacement therapy [42]. No data are available for nadroparin in patients with renal insufficiency.

These results are in accordance with studies on therapeutic dosages, except for dalteparin. Dalteparin is the only LMWH that appears to accumulate when used in a therapeutic dosage, while in studies using prophylactic dosages no accumulation was detected [31–34, 43]. A single-dose study showed reduced elimination of prophylactic dalteparin in patients with renal insufficiency, but apparently clearance is sufficient to prevent accumulation [31–34, 44].

Data on assessment of accumulation on prophylactic nadroparin in renal insufficiency are lacking. Only one multiple-dose study in six patients with CrCl above 30 ml/min and one single-dose study were conducted [45, 46].

Our findings confirm the theory that accumulation seems to depend on the mean molecular weight of LMWHs as shown in Table 3. LMWHs with the lowest molecular weight (enoxaparin, bemiparin, certoparin, and nadroparin) all showed accumulation in a therapeutic dosage and a prophylactic dosage. Tinzaparin (the LMWH with the highest mean molecular weight) has shown not to accumulate in neither therapeutic nor prophylactic dosage. The most likely explanation for this counterintuitive relationship between size and accumulation is that the larger molecules are less dependent on renal clearance [4, 28, 49, 50].

**Table 3:** Accumulation dependency on molecular weight

LMWH	Mean molecular weight (Da) [13, 47]	Accumulation therapeutic	Accumulation prophylactic
Bemiparin	3600	CrCl < 30 ml/min [38]	CrCl < 30 ml/min [38]
Certoparin	3800	CrCl < 30 ml/min [48]	CrCl < 30 ml/min [39]
Nadroparin	4300	Yes^a^ [20]	No conclusion^b^
Enoxaparin	4500	CrCl < 30 ml/min [21–24]	CrCl < 30 ml/min 4 days [37] and 20–50 ml/min 8 days [35]
Dalteparin	6000	CrCl < 30 ml/min after 6 days [32], but not after 3 [43]	No^c^ [31–34]
Tinzaparin	6500	No^d^ [25, 26]	No^d^ [35]

*CrCl* creatinine clearance

^a^Only correlation GFR/anti-Xa activity reported, no specific accumulation limit

^b^Only one multiple dose study in six patients with CrCl above 30 ml/min and one single intravenous dose study [45, 46]

^c^Largest study no lower limit for CrCl^33^

^d^CrCl > 20 ml/min

In patients undergoing renal replacement therapy, it has been shown that many LMWHs are safe to use even in therapeutic dosages [28, 51]. For assessment of accumulation, only a few studies have been conducted. Most of these studies were excluded from this review as they included a single bolus administration, a therapeutic dosage, or no anti-Xa activity measuring at multiple days. Dalteparin showed accumulation in hemodialysis patients if prescribed in a therapeutic dosage, but not in a prophylactic dosage, whereas accumulation was found for prophylactic dosage in peritoneal dialysis patients [19, 40, 52]. Prophylactic dosages of enoxaparin and prophylactic and therapeutic dosages of nadroparin showed no accumulation in hemodialysis and CVVH patients [40, 41, 53, 54]. Tinzaparin accumulation has been found in a therapeutic dose in hemodialysis patients, but in a prophylactic dose, anti-Xa activity returned to baseline in 24 h [52, 55]. In conclusion, for dalteparin accumulation in renal replacement therapy is comparable to accumulation in patients without renal replacement therapy, but in other LMWHs accumulation in renal replacement therapy seems not to depend on mean molecular weight of LMWHs. The mechanism for accumulation in renal replacement therapy is unknown, but the highly negative charge of LMWHs might play a role [52]. More studies are needed to assess LMWH accumulation in patients on renal replacement therapy.

The strength of this review is first that we included only articles that objectively observed accumulation based on anti-Xa activity rather than an accumulation prediction based on the half-life or the time it takes for anti-Xa activity to return to baseline. Second, by including the recent studies on bemiparin and certoparin, this paper confirms the earlier stated theory that accumulation depends on the mean molecular weight of LMWHs.

A limitation might be that we did not include single-dose studies. However, a single-dose study is not suitable for objectively detecting accumulation [28, 32]. A significantly lower clearance or prolonged half-life for a LMWH in a single-dose study does not necessarily indicate that the LMWH accumulates, as is clear from the findings on dalteparin. Furthermore, follow-up of patients in some studies was relatively short (in some studies less than 1 week); however, if LMWHs would accumulate, this would be noticeable already after three dosages. Given the fact that patients tend to be discharged from the hospital within a few days, it is hardly possible to perform clinical studies with longer follow-up.

Another limitation might be that the risk of bias could not be assessed accurately. Considering bias across studies, we feel that a publication bias seems unlikely since both negative and positive outcomes in studies on accumulation have news value, and we included two abstracts of unpublished data. Furthermore, a possible confounder could be the difference in renal insufficiency onset (acute vs chronic) in different studies; however, there is no evidence that this can cause variance in anti-Xa activity.

A major limitation of all studies is that anti-Xa activity rather than hard clinical endpoints were studied. Although a study on hard clinical endpoints in patients with renal insufficiency is probably not feasible due to large numbers needed, it should be taken into account that the correlation between anti-Xa activity and occurrence of bleeding or VTE is not unambiguous [56–60]. The therapeutic and prophylactic target levels of anti-Xa activity are not supported by evidence of trials, but they are rather based on expert opinions [61, 62]. We also found in our review that in six studies that reported clinical outcomes, none of the patients with bleeding had higher anti-Xa activity than patients without bleeding [31–36]. However, the anti-Xa activity is considered to be the best test available to measure LMWH activity and to detect accumulation of LMWHs [4, 63].

In conclusion, for several LMWHs the guidelines that recommend dose reduction for prophylactic use in patients with renal insufficiency are evidence based, except for dalteparin, tinzaparin, and nadroparin. We recommend a dose reduction for prophylactic use of enoxaparin, bemiparin, and certoparin in patients with CrCl below 30 ml/min [24, 28, 29, 38, 39]. Prophylactic dosages of tinzaparin and dalteparin are likely to be safe in patients with renal insufficiency and do not need dose reduction. Studies are needed to assess accumulation of prophylactic dosages of nadroparin and for all LMWHs in patients undergoing renal replacement therapy.

## Electronic supplementary material

ESM 1 (DOCX 51 kb)
