# Supplementary material for: A systematic review on the accumulation of prophylactic dosages of low-molecular-weight heparins (LMWHs) in patients with renal insufficiency
Source: Eur J Clin Pharmacol. 2015 Jun 14;71(8):921–9. doi: 10.1007/s00228-015-1880-5 (PMC4500846; doi:10.1007/s00228-015-1880-5)
Supplement: Supplementary file 1 — (DOCX 51 kb) [file 228_2015_1880_MOESM1_ESM.docx]

Search term as used in Embase:

('low molecular weight heparin'/exp OR (('low molecular weight' NEXT/1 heparin*) OR lmwh* OR adomiparin* OR antixarin* OR ardeparin* OR bemiparin* OR certoparin* OR 'cy 222' OR dalteparin* OR danaparoid* OR deligoparin* OR embolex* OR enoxaparin* OR fondaparinux* OR idrabiotaparinux* OR idraparinux* OR (livaraparin NEXT/1 calcium*) OR minolteparin* OR monoembolex* OR nadroparin* OR parnaparin* OR 'rd 11885' OR reviparin* OR semuloparin* OR sevuparin* OR tafoxiparin* OR tedelparin* OR tinzaparin*):ab,ti) AND ('kidney disease'/exp OR 'kidney function'/exp OR (((kidney* OR renal* OR nephro*) NEAR/6 (disease* OR fail* OR dysfunction* OR impair* OR injur* OR ischaem* OR disorder* OR insufficien* OR function* OR syndrom*)) OR nephropath*):ab,ti) AND ('drug accumulation'/de OR pharmacokinetics/de OR bioaccumulation/de OR (accumulat* OR pharmacokinetic* OR bioaccumulat*):ab,ti)
